# It’s not just the phase: Frequency-dependent tuning of neuronal firing

**DOI:** 10.1371/journal.pbio.3003846

**Published:** 2026-06-24

**Authors:** Ying Yao, Simon Hanslmayr

**Affiliations:** 1 School of Psychology and Neuroscience, University of Glasgow, Glasgow, United Kingdom; 2 Centre for Neurotechnology, University of Glasgow, Glasgow, United Kingdom

## Abstract

This primer discusses a study in PLOS Biology showing that neuronal firing is selectively tuned to oscillatory frequency in human intracranial recordings, complementary to phase tuning, and suggesting an additional dimension in how brain rhythms may organize neural activity.

## Teaser

A new study in *PLOS Biology* shows that neuronal firing is selectively tuned to oscillatory frequency in human intracranial recordings, complementary to phase tuning, suggesting an additional dimension in how brain rhythms may organize neural activity.

**Fig 1:**
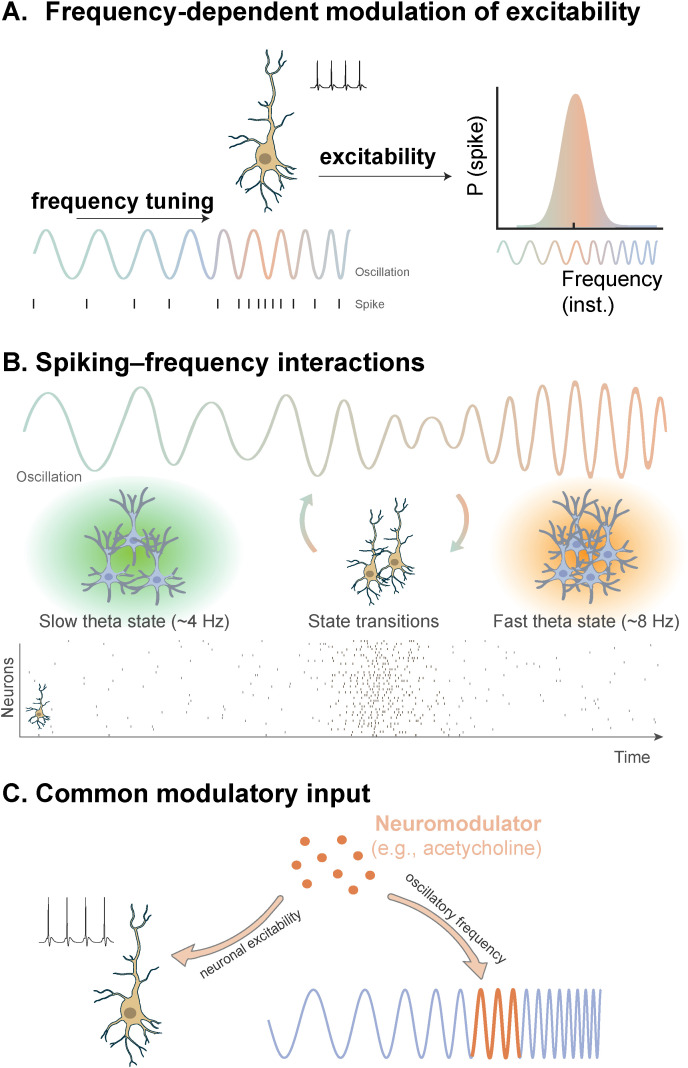
Three possible interpretations of frequency tuning. **(A)** Oscillatory frequency changes in the local field potential (LFP) may directly modulate neuronal excitability, such that neurons that are selectively sensitive to a given frequency increase their firing rate. As a result, neuronal spiking exhibits a frequency-dependent tuning profile, with maximal firing near a preferred frequency. **(B)** Alternatively, the relationship between oscillatory frequency and neuronal firing may be reciprocal. Changes in firing activity of frequency-sensitive neurons may themselves contribute to shifts in LFP frequency, particularly during transitions between network states (e.g., from slow- to fast-theta regimes). In this view, increased spiking may both reflect and participate in state-dependent changes in oscillatory dynamics. **(C)** A third possibility is that both oscillatory frequency and neuronal firing are co-modulated by a common factor, such as neuromodulatory input (e.g., acetylcholine), leading to correlated changes in both without a direct causal link between them. Neuron icon adapted from NIH BioArt Source (bioart.niaid.nih.gov/bioart/424).

Neural activity in the brain is often organized into rhythmic patterns, commonly referred to as neural oscillations, which are reflected in local field potentials (LFPs) and thought to coordinate communication across neural populations [1]. Neurons may preferentially fire at specific moments within an oscillatory cycle (phase tuning) or during oscillations at particular frequencies (frequency tuning). Oscillatory phase has long been implicated in shaping spike timing, but whether fluctuations in oscillatory frequency also relate to neuronal firing has remained unclear. In a recent study published in *PLOS Biology*, Jourahmad and colleagues [2] provide evidence from human intracranial recordings that neuronal firing is associated not only with oscillatory phase but also with moment-to-moment changes in oscillatory frequency, suggesting a complementary dimension of neural dynamics (Fig 1).

A key feature of neural oscillations is that they are inherently non-stationary: their frequencies shift over time rather than remaining fixed. Although the functional significance of these fluctuations remains debated, converging evidence suggests they are closely linked to behavior and cognition. In rodents, theta frequency varies systematically with running speed, potentially preserving phase coding during navigation [3]. In humans, oscillatory slowing has been associated with enhanced working memory capacity [4], while shifts in oscillatory frequency have also been linked to episodic memory processes [5]. Variability in dominant alpha rhythms similarly tracks task demands and time on task [6,7], suggesting that dynamic frequency modulation may represent a general feature of neural activity rather than incidental noise. Yet whether and how these fluctuations relate to neuronal firing has remained largely unresolved.

One reason this question remains unresolved may be methodological. Neural signals are inherently complex, reflecting multiple overlapping rhythms that vary across individuals and over time. To manage this complexity, analyses have often relied on predefined frequency bands, such as theta (4–7 Hz) or alpha (8–12 Hz). While practical and often effective, such approaches can obscure transient frequency fluctuations and impose assumptions about where functionally relevant rhythms should lie. To overcome these limitations, Jourahmad and colleagues [2] adopted a data-driven approach that characterizes oscillatory structure directly from the signal itself. Rather than assigning activity to fixed frequency ranges, the method represents the signal as a combination of oscillatory components and identifies the description that best captures its dynamics. This allows ongoing fluctuations in phase and frequency to be tracked in a way that more closely follows the signal’s intrinsic dynamics.

Using this approach, the authors compared the distribution of frequencies at spike times with that of the ongoing signal, revealing a distinct subset of neurons tuned to particular oscillatory frequencies. These frequency-tuned neurons showed little overlap with phase-tuned neurons, suggesting that these may represent mathematically dissociable forms of modulation. Nevertheless, phase and frequency remain closely linked properties of oscillatory activity, since changes in frequency necessarily alter phase progression and may consequently influence phase alignment within and across neural networks [8], indicating that these dimensions are not physiologically independent.

Importantly, frequency tuning was most prominent at lower frequencies, particularly below 10 Hz, which are often associated with large-scale coordination and memory-related processes. This effect could not be explained simply by the dominance of low-frequency activity in the signal, as neurons responded selectively within relatively narrow frequency ranges. This preference for lower frequencies may therefore be functionally significant rather than incidental. Lower-frequency oscillations are thought to support long-range communication across distributed neural populations, consistent with communication-through-coherence accounts in which inter-regional synchrony facilitates information exchange [9]. The longer temporal windows associated with slower oscillations may additionally promote spike coordination and synaptic plasticity mechanisms linked to memory formation [5]. At the same time, this apparent low-frequency bias should be interpreted cautiously, as slower oscillations may be more readily detected than local high-frequency dynamics.

Alongside this low-frequency bias, the authors also reported bidirectional modulation within individual neurons, with firing increasing at certain frequencies but decreasing at others, suggesting interactions that extend beyond a simple tuning rule. One possibility is that oscillatory frequency regulates the temporal scale over which neural activity is integrated. Faster oscillations may support shorter integration windows and tighter spike co-firing, potentially facilitating temporally precise plasticity mechanisms during memory formation [5], whereas slower rhythms may extend integration windows and increase the amount of information maintained within a cycle, as suggested by studies linking slower theta frequencies to enhanced working memory capacity [4]. Frequency fluctuations may also contribute to coordination across spatial scales, since large-scale and local networks often operate at different intrinsic frequencies and may transiently adjust their frequencies to facilitate phase alignment and information exchange across regions [8]. However, the direction of causality remains unresolved. Frequency fluctuations may actively shape neural coordination. Conversely, shifts in neuronal firing and network interactions may themselves contribute to changes in oscillatory frequency—perhaps during transitions between network states—or both may be jointly driven by a third factor, such as neuromodulators like acetylcholine, which are known to regulate theta dynamics [10]. Consistent with this ambiguity, preferred firing frequencies often lie near, but not exactly at, dominant LFP frequencies, which reflect coordinated population-level activity across local neural networks, suggesting tuning may in some cases reflect broader state-dependent network dynamics rather than purely frequency-based coding [2].

These findings raise broader questions about what frequency tuning may reflect. Because the present analyses were limited to resting-state activity, an important next step will be to determine whether frequency tuning plays a functional role during behavior and cognitive processing. Future work should test this explicitly in task-based settings and, ideally, through causal manipulations of oscillatory frequency, including stimulation, neuropharmacological interventions, or behavioral modulation, while measuring resulting changes in neuronal spiking and inter-regional coordination. Such approaches are beginning to emerge, from stimulation studies demonstrating entrainment of single-neuron activity in nonhuman primates [11,12], to recent work exploring how externally imposed rhythms interact with ongoing oscillatory dynamics. These manipulations may help determine whether oscillatory frequency merely tracks network state or can actively tune neuronal firing and large-scale neural coordination.

Overall, the study suggests that brain rhythms may influence neuronal activity in more diverse and dynamic ways than previously appreciated, extending beyond phase-based accounts of spike timing. Whether frequency tuning reflects a coding mechanism, a marker of state transitions, or both remains unresolved. Nevertheless, considering phase and frequency together may provide a richer framework for understanding—and potentially manipulating—how oscillatory dynamics coordinate neural activity across time and space.

## Abbreviation:

LFP: local field potential
